# Fungal small RNA: unveiling the breakthroughs and promising applications

**DOI:** 10.3389/ffunb.2025.1670120

**Published:** 2026-04-20

**Authors:** Shuquan Sun, Junjian Situ, Chuan Gao, Yixuan Chai, Changsheng Lv, Xiangyu Ma, Yuanyuan Zhang

**Affiliations:** 1 School of Environmental Engineering, Yellow River Conservancy Technical University, Kaifeng, Henan, China; 2 Kaifeng Engineering Technology Research Center of Aquatic Environmental Pollution Monitoring, Kaifeng, Henan, China; 3 Henan Engineering Technology Research Center of Green Coating Materials, Kaifeng, Henan, China; 4 Department of Plant Pathology, South China Agricultural University, Guangzhou, Guangdong, China

**Keywords:** biocontrol, biogenesis mechanisms, fungi, RNA interference (RNAi), small RNA

## Abstract

Small RNAs (sRNAs) are abundant endogenous non-coding RNAs in eukaryotic organisms that regulate gene expression by binding to their target mRNAs either completely or partially. The rapid advancements in fungal sRNA research in recent years have significantly expanded our understanding of their biogenesis, functional mechanisms, and roles in fungal biology. Unlike previous reviews that predominantly focus on the intracellular biogenesis and regulatory networks of fungal sRNAs within fungal cells, this review uniquely bridges fungal sRNA molecular biology with plant pathology by centering on the bidirectional cross-kingdom RNAi trafficking between fungi and plants. We provide a comprehensive overview of fungal sRNA types, especially novel subtypes identified in recent studies, the key protein factors involved in their biogenesis, and the molecular mechanisms governing their intracellular functions. Additionally, we conduct an in-depth analysis of the trafficking routes of fungal sRNAs into host plants, their targeted interference with plant immune signaling cascades, and the reciprocal regulation of fungal physiology by plant-derived sRNAs. Finally, we discussed the potential applications of fungal sRNAs in biotechnology and pathogen control, particularly in the development of host-induced gene silencing (HIGS)/spray-induced gene silencing (SIGS)-based crop protection strategies. This work not only serves as a valuable reference for future studies on fungal sRNAs but also highlights the translational potential of cross-kingdom RNAi in plant–fungal pathosystems, filling critical gaps in existing literature.

## 1 Introduction

Small RNAs (sRNAs) are non-coding single-stranded RNA molecules, typically ranging from 20 to 40 nucleotides in length. They regulate gene expression through chromatin modifications, post-transcriptional mRNA degradation, or translational inhibition, playing a crucial role in eukaryotic growth, development, and immune responses (Gabriel et al., 2021). Due to their fundamental regulatory functions, sRNAs have become a central focus in modern molecular biology. In mammals, sRNA biogenesis has been closely linked to cancer and other pathological conditions, whereas in plants, sRNAs primarily mediate responses to biotic and abiotic stresses. Fungi, as widely distributed organisms with significant ecological and industrial importance, have also been a growing subject of sRNA research. The increasing availability of fungal genome sequences, coupled with advances in high-throughput sequencing and computational biology, has propelled significant progress in the characterization and functional analysis of fungal sRNAs. These studies have shed light on the diverse roles of sRNAs in fungal development, pathogenicity, stress adaptation, and interactions with host organisms.

Notably, most existing reviews on fungal sRNAs have centered narrowly on intracellular biogenesis pathways and intraspecific regulatory networks, with limited attention to the bidirectional cross-kingdom RNA interference (RNAi) dynamics between fungi and their plant hosts—a gap that hinders the translation of basic research into agricultural applications (Guo et al., 2019). By contrast, this review distinguishes itself by integrating fungal sRNA molecular biology with plant pathology, placing a core emphasis on cross-kingdom sRNA trafficking mechanisms and their regulatory roles in plant–fungal interactions. This review synthesizes the current understanding of fungal sRNAs by summarizing the major sRNA classes identified to date, the protein factors implicated in sRNA biogenesis, and the molecular mechanisms governing their activity. Additionally, we discuss the broader implications and potential applications of fungal sRNAs, particularly in the development of RNAi-based strategies for plant disease control.

## 2 Types of fungal sRNAs

**Figure 1:**
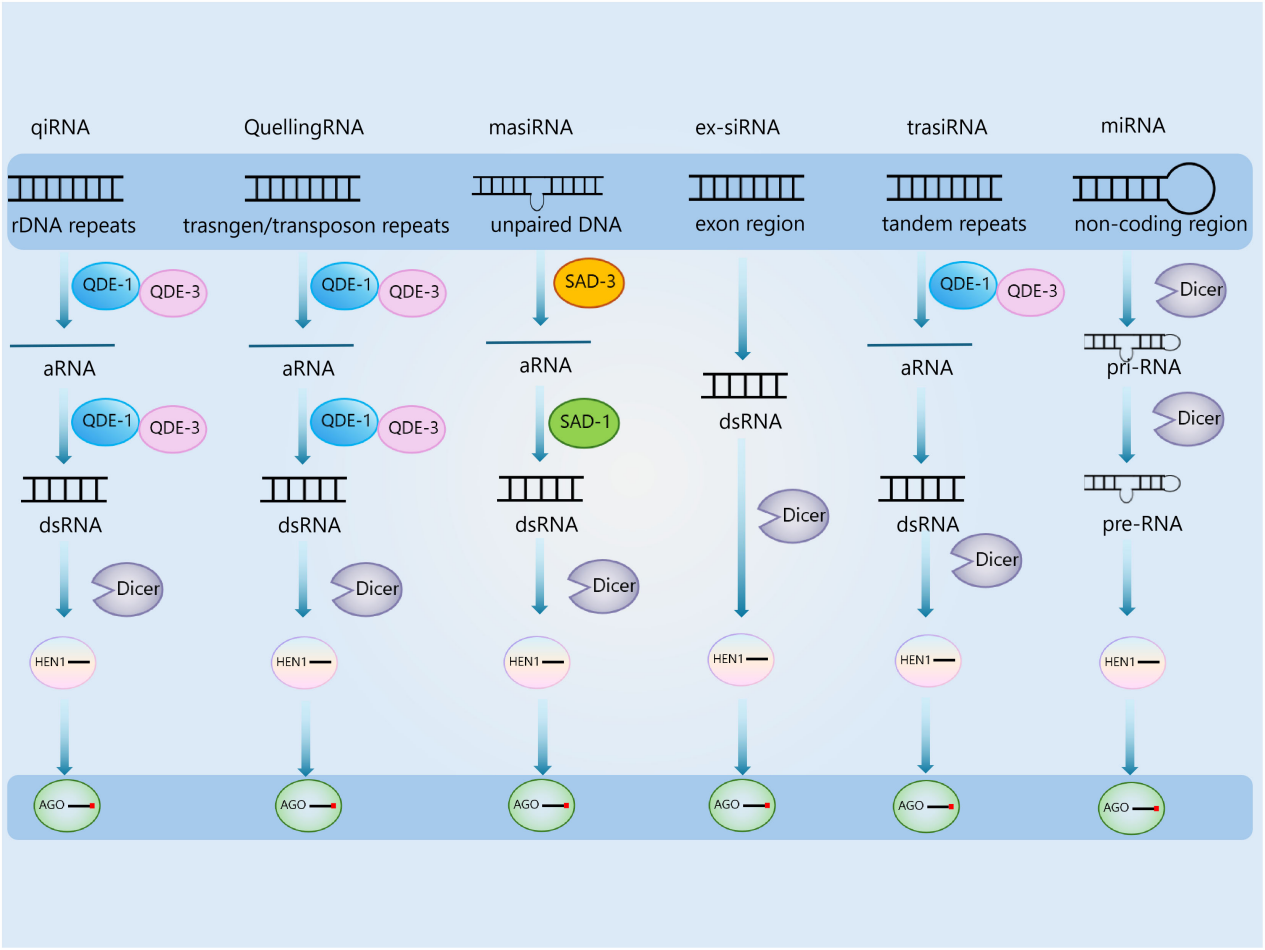
Fungal small RNA mechanism. This diagram illustrates the synthesis of six major sRNA types in fungi, each derived from distinct genomic regions and processed by specific protein machineries: qiRNA, quellingRNA, masiRNA, ex-siRNA, trasirNA, and miRNA.

Currently, sRNAs are classified into three major categories: microRNAs (miRNAs), small interfering RNAs (siRNAs), and PIWI-interacting RNAs (piRNAs) (Chen and Rechavi, 2022). Among eukaryotes, siRNAs (Hannon, 2002) and miRNAs (Lee et al., 1993) are the most commonly studied, initially identified in *Caenorhabditis elegans* and subsequently discovered in various plant and animal species. Recent studies have also identified siRNAs in fungi (**Table 1**), including the model fungus *Neurospora crassa* and plant pathogenic fungi such as *Magnaporthe oryzae* (Nakayashiki, 2005), *Fusarium graminearum* (Hou et al., 2025), and *Botrytis cinerea* (He et al., 2023) (**Figure 1**).

**Table 1:** Function of distinct fungal small RNAs.

Fungal species	Small RNA type	Function/Mechanism	Host	Reference
*Neurospora crassa*	miRNA-like RNAs (milRNAs)	Regulate gene silencing/heterochromatin formation and developmental transitions	–	Lee et al. (2010)
*Neurospora crassa*	qiRNA	Induced by DNA damage/associates with QDE-2 to mediate post-transcriptional gene silencing	–	Lee et al. (2009)
*Botrytis cinerea*	milRNAs	Suppress host plant immunity by targeting defense-related mRNAs in Arabidopsis	*Arabidopsis thaliana*/tomato	Weiberg et al. (2013)
*Fusarium graminearum*	milRNAs	Modulate fungal virulence and secondary metabolism during wheat infection	Wheat	Hou et al. (2025)
*Fusarium graminearum*	ex-siRNAs	Exert stage-specific functions during the late phase of sexual development; regulate ascospore discharge	Wheat	Hou et al. (2025); Zeng et al. (2024)
*Fusarium graminearum*	trasiRNA	Regulate sexual reproduction	Wheat	Hou et al. (2025)
*Cryptococcus neoformans*	siRNA	Control transposon silencing and genome stability under stress conditions	–	Yadav et al. (2024)
*Magnaporthe oryzae*	ex-siRNAs	Transfer to host rice cells to suppress immunity by targeting rice PRR genes	Rice	Gowda et al. (2010); Raman et al. (2017)
*Magnaporthe oryzae*	tRFs	Enriched in appressoria and spores/implied to regulate protein synthesis	Rice	Cole et al. (2009); Nunes et al. (2011)
*Aspergillus flavus*	milRNAs	Regulate aflatoxin biosynthesis and oxidative stress response	–	Bai et al. (2015)
*Fusarium oxysporum* f. sp. *cubense*	milRNAs (milR-87)	Promote virulence by silencing glycosyl hydrolase-encoding genes	Banana	Li et al. (2022), Li et al, 2023
*Trichoderma reesei*	milRNAs	Regulate growth and cellulase induction	–	Kang et al. (2013)
*Mucor circinelloides*	ex-siRNAs	Regulate fungal developmental processes	–	Nicolas et al. (2010)
*Schizosaccharomyces pombe*	rasiRNA	Associated with heterochromatin formation and genome stability	–	Reinhart and Bartel (2002)

Expanding research on fungal siRNAs has led to the discovery of multiple novel siRNA types. In *N. crassa*, a unique class of siRNA known as qiRNA (QDE-2-interacting small RNA) has been identified (Lee et al., 2009). These qiRNAs, typically 20–21 nucleotides in length, are induced by DNA damage and are associated with the DNA-dependent RNA polymerase QDE-2, mediating post-transcriptional gene silencing through interaction with this enzyme (Lee et al., 2009). Similarly, in *Mucor circinelloides*, *Trichoderma atroviride*, and *F. graminearum*, another class of sRNAs called exonic-siRNAs (ex-siRNAs) has been reported (Carreras-Villasenor et al., 2013; Nicolas et al., 2010; Son et al., 2017). Ex-siRNAs have been proposed to regulate the target gene expression involved in fungal developmental processes in *M. circinelloides* and *T. atroviride* (Carreras-Villasenor et al., 2013; Nicolas et al., 2010; Son et al., 2017) and to exert stage-specific functions, particularly during the late phase of sexual development in *F. graminearum* (Son et al., 2017; Zeng et al., 2024).

Another category of fungal sRNAs, repeat-associated small interfering RNAs (rasiRNAs), has been identified in *Schizosaccharomyces pombe*. These sRNAs exhibit variable lengths across different fungal species, often resembling or slightly exceeding the size of canonical Dicer-dependent siRNAs or miRNAs (Reinhart and Bartel, 2002). Further studies are required to determine their presence in other fungal species and the specific Dicer homologs responsible for their production.

High-throughput sequencing of sRNAs in *M. oryzae* has revealed the presence of tRNA-derived RNA fragments (tRFs). These sRNAs originate from either the 5′ or 3′ ends of tRNAs and are generated via cleavage near or within the anticodon loop, forming two distinct fragments (Cole et al., 2009; Jochl et al., 2008; Nunes et al., 2011). Notably, tRFs are significantly more abundant in *M. oryzae* appressoria and spores than in hyphae, suggesting a regulatory role in protein synthesis. This trend aligns with the high demand for intact tRNAs in hyphal growth, which contrasts with the regulatory requirements of appressoria and spores (Nunes et al., 2011). Small interfering RNAs associated with tandem repeat-induced sexual silencing (trasiRNAs) in tandem repeats were reported in *F. graminearum*. Tandem repeat-induced sexual silencing (TRISS) is a unique RNAi pathway and uses a distinct combination of components from the RNAi machinery involved in quelling and MSUD pathways during fungal sexual stages (Gowda et al., 2010; Son et al., 2017). The growing body of research on fungal sRNAs highlights their complex regulatory roles and underscores the need for further investigations to elucidate their biogenesis, functional significance, and potential applications in biotechnology and disease control.

Additionally, miRNA-like RNAs (milRNAs) have been characterized based on their resemblance to plant and animal miRNAs, first reported in *N. crassa* (Lee et al., 2010). Classical investigations on *N. crassa* have indicated that milRNA precursors (pri-milRNAs) are initially cleaved by Dcl1, leading to the formation of hairpin-structured pre-milRNAs. Subsequently, these pre-milRNAs undergo secondary cleavage by Dcl2 to generate mature milRNAs. In dcl1/dcl2 double-knockout mutants, the detectable signals of milRNAs are entirely abolished. Verified through Northern blot analysis, this conclusion has long been recognized as the standard model for milRNA biogenesis, and subsequent research on *Aspergillus nidulans* has also corroborated this mechanism (Tabara et al., 2021). Nevertheless, investigations on *Penicillium chrysogenum* have presented counterexamples. Regarding milRNA-Pc1 in *P. chrysogenum*, its accumulation level merely decreases by 30% in dcl1 single-knockout mutants and remains constant in dcl2 single-knockout mutants (Dahlmann and Kück, 2015). Complete depletion of milRNA-Pc1 is only witnessed in dcl1/dcl2 double-knockout mutants (Tabara et al., 2021). Furthermore, its precursor is a linear single-stranded RNA rather than a hairpin-structured transcript, suggesting the existence of a Dicer-independent biogenesis pathway (Xue et al., 2012).

Initial investigations on *N. crassa* were dependent on short-read sequencing (Illumina platform) (Tabara et al., 2021). This approach was unable to comprehensively capture the complete structure of milRNA precursors, resulting in the misapprehension that the predominant Dicer-dependent pathway was the sole pathway. Conversely, research on *P. chrysogenum* combined bioinformatic prediction with mutant validation, clearly differentiating between Dicer-dependent and Dicer-independent subtypes, thereby generating more reliable findings (Dahlmann and Kück, 2015). In light of the most recent research advancements and prevalent issues in this domain, the study of fungal small RNA presents numerous conflicting results attributable to species specificity, technical constraints, and disparities in experimental design. Additionally, the experimental robustness of certain core conclusions has not been fully substantiated.

## 3 sRNA-associated proteins

**Figure 2:**
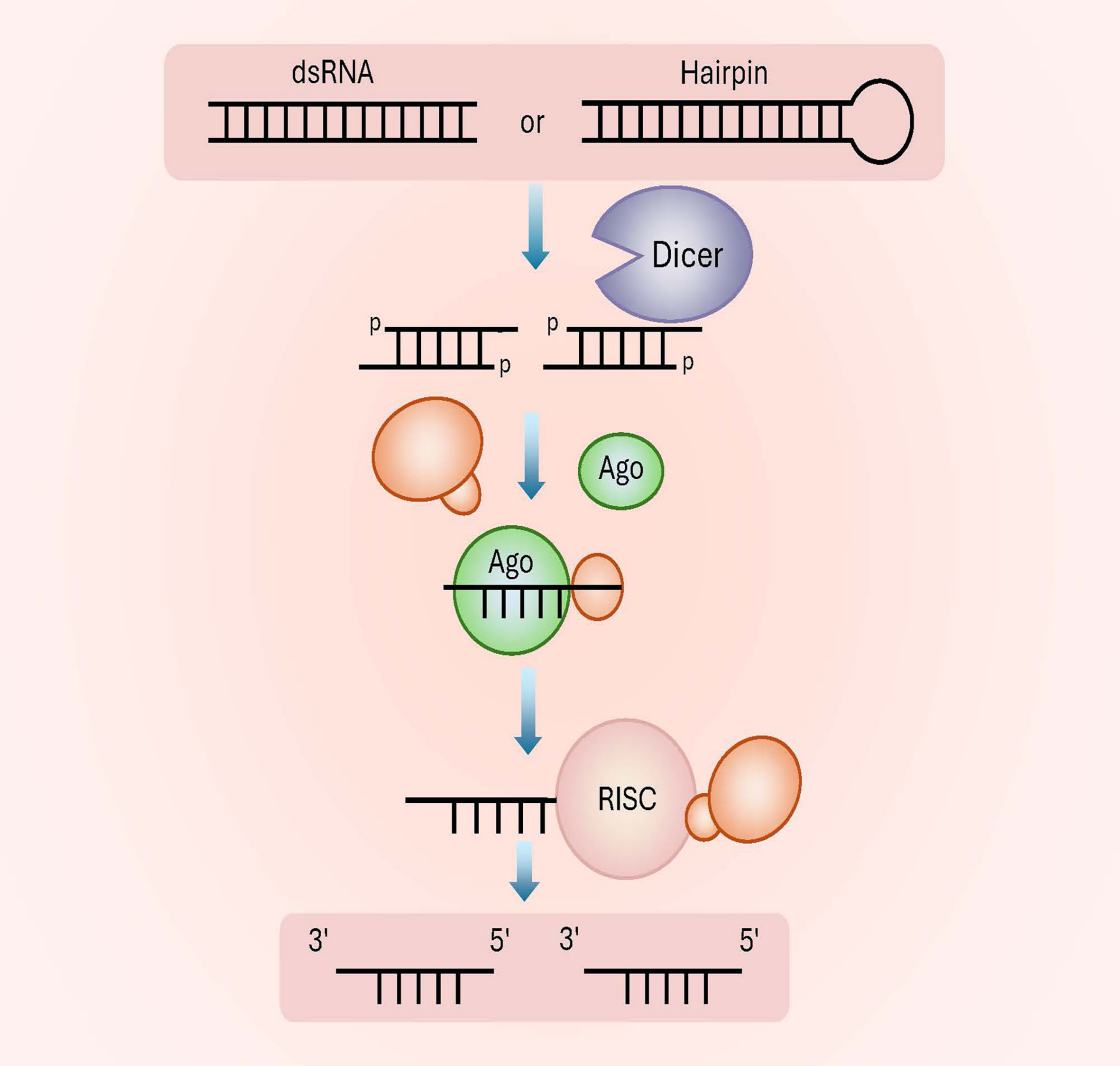
Fungal small RNA-associated protein. This diagram outlines the conserved process of sRNA maturation and RISC formation in eukaryotes. sRNA precursor: starts with either double-stranded RNA (dsRNA) or hairpin-structured RNA (transcribed from non-coding regions). Dicer cleavage: The precursor is processed by Dicer (RNase III enzyme) into 21–24 nt sRNA duplexes (each strand carries a 5′ phosphate group, labeled “p”). AGO loading: One strand of the sRNA duplex (the guide strand) is loaded into an Argonaute (AGO) protein; the passenger strand is degraded. RISC formation: The AGO–sRNA complex assembles with auxiliary proteins to form the functional RISC, which mediates target RNA silencing (cleavage or translational repression) via complementary base pairing.

The functional mechanisms of sRNAs are highly complex and vary across species and even among different sRNAs within the same organism. However, a conserved core process can be identified (**Figure 2**). The primary proteins associated with fungal sRNAs include Dicer, Argonaute, and RNA-dependent RNA polymerase (Rdrp) (Kwasiborski et al., 2022). Extensive functional studies on these protein families in fungi have led to significant research advancements (**Table 2**).

**Table 2:** Fungal small RNA-associated proteins.

Protein name	Protein type	Function	Fungal species	Involvement in sRNA biosynthesis pathways	Reference
DCL1 DCL2	Dicer-like protein	DCL1 cleaves pri-milRNA to pre-milRNA; DCL2 mediates secondary cleavage of pre-milRNA to mature milRNA; DCL2 processes 30–130 nt dsRNA into 23 nt sRNAs	*Neurospora crassa*	Dicer-dependent milRNA biogenesis; siRNA biogenesis (quelling pathway)	Catalanotto et al. (2004); Lee et al. (2010); Tabara et al. (2021)
Dicer-2	Dicer-like protein	Solely responsible for siRNA production; mediates antiviral silencing and vsRNA biogenesis	*Magnaporthe oryzae*	*Cryphonectria parasitica*	Andika et al. (2019); Kadotani et al. (2004)
Dicer-1 Dicer-2	Dicer-like protein	Dicer-1 regulates hyphal growth; Dicer-2 drives sRNA production and conidial germination	*Mucor circinelloides*	*Fusarium graminearum*	Nicolas et al. (2007); Wang et al. (2022)
CaDcr1	Dicer-like protein	Involved in siRNA production and ribosomal/spliceosomal RNA maturation	*Candida albicans*	siRNA biogenesis; RNA maturation (non-canonical Dicer function)	Weinberg et al. (2011)
QDE-2 (AGO)	Argonaute protein	Forms a RISC complex with QIP; binds qiRNA and mediates gene silencing; required for milR-87 biogenesis in *F. oxysporum*	*Neurospora crassa*	*Fusarium oxysporum*	Lee et al. (2009); Maiti et al. (2007)
Ago1 Ago2	Argonaute protein	Ago1 regulates conidial morphogenesis; Ago2 controls germ tube elongation and mycotoxin synthesis	*Fusarium oxysporum*	*Fusarium graminearum*	Song et al. (2018); Wang et al. (2022)
Agl2	Argonaute-like protein	Mediates antiviral defense and viral RNA recombination	*Cryphonectria parasitica*	Antiviral RNA silencing pathway	Sun et al. (2009)
QDE-1 (RdRP-6)	RNA-dependent RNA polymerase	Synthesizes dsRNA from aberrant RNAs; interacts with RPA to link RNAi and DNA replication	*Neurospora crassa*	dsRNA precursor synthesis for siRNA biogenesis; quelling pathway	Cogoni and Macino (1999a); Nolan et al. (2008a)
Rdrp-1 Rdrp-2	RNA-dependent RNA polymerase	Rdrp-1 initiates forward transgene-induced silencing; Rdrp-2 amplifies secondary siRNAs	*Mucor circinelloides*	Initiation and amplification of RNA silencing signals	Piombo et al. (2023); Sun et al. (2009)
QIP	Exonuclease	Cleaves and degrades the passenger strand of siRNAs to activate RISC	*Neurospora crassa*	RISC activation in siRNA-mediated silencing	Maiti et al. (2007)

### 3.1 Dicer

Dicer, a nuclease belonging to the RNase III family, plays a crucial role in cleaving double-stranded RNA (dsRNA) into 21–25 nucleotide sRNAs. In fungi, one or two Dicer proteins have been identified, with some species exhibiting non-redundant functional specialization. In *N. crassa*, both Dicer homologs participate in RNA silencing (Catalanotto et al., 2004), with DCL-2 processing dsRNA substrates of 30–130 nucleotides, generating predominantly 23-nucleotide sRNAs (Tabara et al., 2021). In contrast, in *M. oryzae*, Dicer-2 is solely responsible for siRNA production (Kadotani et al., 2004), likely due to transcriptional repression and protein specialization (Kadotani et al., 2008). Similarly, in *M. circinelloides*, Dicer-1 regulates hyphal growth, whereas Dicer-2 drives sRNA production, highlighting their distinct roles (Nicolas et al., 2007).

In *Saccharomyces cerevisiae* and the pathogenic fungus *Candida albicans*, Dicer exhibits structural divergence compared to other fungi. While a typical fungal Dicer contains two RNase III domains, *S. cerevisiae* and *C. albicans* possess only one, yet their RNase III active sites remain highly conserved (Weinberg et al., 2011). Additionally, a double-stranded RNA-binding domain (dsRBD) is located downstream, with another dsRBD domain present at the C-terminal region (Drinnenberg et al., 2009). In *C. albicans*, Dicer is involved in both siRNA production and developmental regulation (Bernstein et al., 2012). In *Cryphonectria parasitica*, Dicer-2 is essential for antiviral RNA silencing and viral small RNA (vsRNA) production, whereas Dicer-1 is dispensable (Andika et al., 2019). In *Trichoderma atroviride*, Dicer-1 and Dicer-2 regulate distinct biological processes, including reproductive development and vegetative growth (Carreras-Villasenor et al., 2013). However, the precise regulatory mechanisms governing these functions remain to be elucidated. Studies on *B. cinerea*, a pathogenic fungus, have demonstrated that the simultaneous knockout of both Dicer genes results in mutants incapable of producing sRNAs, leading to significantly reduced virulence against *Arabidopsis thaliana* and tomato (Cai et al., 2019). Additionally, *A. thaliana* mutants lacking Argonaute-1 exhibit decreased susceptibility to *B. cinerea*. This suggests that during infection, *B. cinerea* secretes sRNAs that interfere with host sRNA pathways, hijacking the plant’s RNAi machinery via Argonaute-1 to selectively silence immune-related genes (He et al., 2023). These findings indicate that fungal RNAi may serve as a higher-order regulatory mechanism in plant–pathogen interactions (Weiberg et al., 2013). This study has established a direct link between sRNAs and fungal pathogenicity, marking a transition in sRNA research from molecular regulatory models to practical applications in biological control. In *Coniothyrium minitans*, single knockouts of Dicer-1 or Dicer-2 resulted in mutants exhibiting markedly decreased growth rates and a weakened capacity to parasitize the sclerotia of *Sclerotinia sclerotiorum* when compared with wild-type strains. However, these mutants displayed markedly higher sporulation rates and increased production of antifungal compounds that inhibit *S. sclerotiorum* hyphal growth (Zeng, 2012). Interestingly, Dicer-2 was found to play a key role in promoting the accumulation of exogenous viruses within the host (Johnson et al., 2022). In *F. graminearum*, Dicer-2 has been implicated in conidial germination and germ tube formation and is also involved in the regulation of mycotoxin synthesis (Wang et al., 2022). Similarly, in *F. oxysporum*, both single and double knockouts of the two Dicer genes revealed that they are essential for the regulation of vegetative growth, development, and reproduction of hyphae (Song et al., 2018). In *Metarhizium anisopliae*, single and double knockouts of Dicer-1 and Dicer-2 did not lead to noticeable differences in colony morphology, hyphal structure, growth rate, or stress resistance compared to the wild-type strain (Meng et al., 2017a). Moreover, the range of upregulation for sporulation-related genes was narrower in the Dicer-2 mutant, and miRNA expression levels were significantly downregulated, indicating that miRNA biogenesis in *M. anisopliae* may be dependent on Dicer-2 (Meng et al., 2017b). Across multiple fungal species, Dicer plays a crucial role in sRNA biogenesis, growth, development, and reproduction (Dahlmann and Kück, 2015; Melet et al., 2025; Meng et al., 2017a). However, the specific biological functions of Dicer proteins and the extent of functional redundancy between Dicer homologs vary significantly among fungi (Melet et al., 2025). Further research is required to elucidate these functions across diverse fungal taxa, investigating their evolutionary relationships and potential coevolution with fungal sRNA pathways. The growing body of research underscores the essential role of sRNAs and Dicer in fundamental biological processes, including fungal development, immunity, and stress responses. Most current studies determine Dicer function by comparing phenotypic, transcriptomic, and sRNA profiles before and after gene knockout. However, to fully unravel the mechanisms underlying Dicer-mediated regulation, future research should focus on identifying interacting proteins and deciphering the broader regulatory networks governing sRNA pathways.

Mutating Dicer genes is a standard method to confirm sRNA biogenesis dependence (Melet et al., 2025). However, most fungi encode multiple Dicer paralogs with functional redundancy. *FgDCL1* and *FgDCL2* genes were partly functionally redundant in ascospore discharge and perithecium-specific milRNA generation in *F. graminearum*, and these perithecium-specific milRNAs play potential roles in sexual development. Robust experiments require generating double/triple knockout mutants to completely abrogate sRNA biogenesis (Hou et al., 2025; Son et al., 2017).

### 3.2 Argonaute

Argonaute (Ago) proteins were initially discovered in *A. thaliana* and later identified as key components of RNA silencing pathways (Grishok et al., 2001; Tabara et al., 1999). Ago proteins primarily mediate RNA silencing by participating in the formation of the RNA-induced silencing complex (RISC), which facilitates the degradation or translational repression of target RNAs (Hammond et al., 2000, Hammond et al., 2001). The critical role of Ago proteins in sRNA-mediated gene silencing was firmly established by Höck and Meister (2008) and Hutvagner and Simard (2008), who demonstrated that Ago is indispensable for the execution of RNAi mechanisms across diverse cellular processes.

Despite extensive research on Dicer proteins, studies on fungal Ago proteins remain relatively underdeveloped. However, investigations into *S. pombe* have provided key insights into the role of Ago in the fungal RNAi pathway. In *S. pombe*, Ago is involved in both transcriptional and post-transcriptional gene silencing, playing a crucial role in the regulation of heterochromatin formation and gene expression at centromeric regions and mating-type loci (Calo et al., 2012). One of the most well-characterized complexes involving Ago in *S. pombe* is the RNA-induced initiation of transcriptional gene silencing (RITS) complex, which consists of Ago1, Chp1, Tas3, and centromeric siRNAs (Sun et al., 2020). This complex is essential for heterochromatin-mediated gene silencing at centromeric loci (Buker et al., 2007; Sun et al., 2020).

In addition to RITS, *S. pombe* possesses an Ago-associated complex known as the Argonaute siRNA chaperone (ARC), which includes Ago1, Arb1, and Arb2 (Buker et al., 2007). This complex plays a fundamental role in histone H3 lysine 9 methylation, heterochromatin assembly, and siRNA biogenesis. Notably, while most siRNAs in the RITS complex are single-stranded, those associated with ARC are predominantly double-stranded (Sun et al., 2020). This distinction suggests that Arb1 and Arb2 may function to inhibit the release of the siRNA lagging strand from Ago1. Experimental evidence supports this hypothesis, as purified Arb1 has been shown to inhibit the slicer activity of Ago1 *in vitro* (Sun et al., 2009a). Furthermore, catalytically inactive Ago1 has been found to associate predominantly with double-stranded siRNAs, indicating that the conversion of double-stranded siRNAs into single-stranded forms is a prerequisite for effective heterochromatin formation (Buker et al., 2007; Sun et al., 2020). The Arb proteins appear to play a pivotal role in facilitating this conversion, thereby ensuring proper chromatin regulation and gene silencing (Calo et al., 2012).

In *C. parasitica*, four Ago proteins have been identified; however, only Agl2 (*Ago-like 2*) has been implicated in antiviral defense and RNA recombination (Sun et al., 2009). This finding underscores the critical role of Ago genes in RNA silencing-based antiviral mechanisms and viral RNA recombination, providing further evidence for how virus-encoded RNA silencing suppressors inhibit the transcriptional activation of RNA silencing components in fungi. In *C. albicans*, a single Ago gene has been identified, exhibiting significant structural differences compared to Argonaute proteins found in other fungi and higher eukaryotes. Knockout studies have shown that *C. albicans* Ago is dispensable for growth and development, as its deletion does not result in noticeable phenotypic changes (Iracane et al., 2024). In *M. circinelloides*, only one of the three Ago genes, *ago-1*, has been shown to participate in RNAi during vegetative growth, transgene-induced RNA silencing, and the accumulation of various endogenous exon-derived small RNAs (esRNAs) (Cervantes et al., 2013). Type I and II ex-siRNAs bind to Ago-1 to regulate the accumulation of mRNAs encoding target proteins, whereas type III ex-siRNAs, although not directly associating with Ago-1, require its presence for their biogenesis. This suggests a complex and multilayered regulatory mechanism governing ex-siRNA biosynthesis. Functional analyses of *ago-1* mutants in *M. circinelloides* indicate that its absence leads to impaired vegetative development and increased autolysis under nutrient stress conditions, suggesting that *ago-1* plays a role in environmental stress response. These findings highlight the ability of a single Ago protein to mediate the production of distinct esRNAs via different pathways, thereby expanding the regulatory potential of RNAi in endogenous gene expression (Cervantes et al., 2013). In *F. graminearum*, Ago-2 has been shown to influence germ tube elongation (Wang et al., 2022), whereas in *F. oxysporum*, Ago-1 is associated with conidial morphogenesis, and Ago-2 is linked to hyphal development. Both proteins appear to suppress conidiation in early growth stages while promoting hyphal extension (Song et al., 2018). In *M. anisopliae*, Ago-1 and Ago-2, like Dicer proteins, do not impact colony morphology, hyphal structure, growth rate, or stress resistance. However, they are involved in regulating sporulation (Meng et al., 2017b). Notably, the deletion of Argonaute genes in *M. oryzae* leads to a significant reduction in sRNA levels and decreased pathogenicity, emphasizing their role in fungal virulence (Raman et al., 2017). Collectively, these findings establish that Ago proteins are essential components of RNA silencing, playing critical roles in fungal growth, development, and stress response. However, despite their importance, distinct phenotypic alterations due to Ago depletion have not been widely observed across fungal species, with the exception of *F. oxysporum* (Li et al., 2022). This could be attributed to the presence of multiple Ago paralogs that exhibit functional redundancy, allowing the loss of one Ago protein to be compensated for by another. Alternatively, specific Ago proteins may play pivotal roles in certain regulatory pathways, with other Ago homologs adapting to maintain cellular homeostasis. Research on Ago proteins in fungi remains in its early stages, and further investigations are needed to elucidate their precise biological functions and functional divergence across different fungal species. Understanding the interplay between Ago-mediated RNAi mechanisms and fungal adaptation may provide novel insights into fungal pathogenesis, stress tolerance, and potential biotechnological applications.

### 3.3 Rdrp proteins

The Rdrp synthesizes complementary RNA strands using RNA as a template, playing a critical role in amplifying RNA silencing signals in eukaryotic RNAi. Rdrps are classified into two primary categories: those responsible for viral genome replication and those found in eukaryotes, which function in RNA silencing by generating dsRNA. The first active Rdrp was isolated from tomato (Schiebel et al., 1998). In *N. crassa*, RNA silencing requires the participation of QDE-1 (quelling-deficient-1), also referred to as Rdrp-6, which shares sequence similarity with tomato Rdrp genes (Cui et al., 2022). QDE-1 is a 1,402-amino acid protein lacking signal peptides and transmembrane regions, suggesting that it functions as a hydrophilic intracellular protein, as indicated by hydropathy analysis (Cogoni and Macino, 1999a). Subsequent studies confirmed that QDE-1 exhibits Rdrp activity (Tijsterman et al., 2002), making it the only Rdrp with an observed crystal structure to date (Salgado et al., 2006). In *S. pombe*, a single Rdrp gene encodes an Rdrp protein involved in centromere function (Volpe et al., 2002) as well as the establishment and maintenance of heterochromatin (Sugiyama et al., 2005). Meanwhile, in *M. circinelloides*, Rdrp-1 is primarily responsible for generating dsRNA during forward transgene-induced silencing, although it does not significantly contribute to secondary siRNA production (Nicolas et al., 2010). Conversely, Rdrp-2 appears to regulate secondary generation of secondary siRNAs, playing a crucial role in effective dsRNA formation and forward transgene-induced silencing (Sun et al., 2009). In *T. atroviride*, Rdrp-3 has been identified as a key regulator of reproductive development (Carreras-Villasenor et al., 2013). Similarly, in *F. oxysporum*, both Rdrp-2 and Rdrp-3 are involved in conidial morphogenesis and reproductive processes (Song et al., 2018). Evolutionary analysis of 161 *Rdrp* genes from animals, plants, and fungi suggests that the ancestral eukaryotic cell contained only three types of Rdrps, which diversified over evolutionary time. Rdrpβ (encompassing animal and fungal Rdrp-3) and Rdrpα (including plant Rdrp-3 and fungal Rdrp-6) exhibit minimal homology, implying potential functional divergence during evolution (Zong et al., 2009). Beyond its established role in RNAi, Rdrp may also be linked to DNA replication. It was found that Rdrp protein QDE-1, which is responsible for post-transcriptional gene silencing in *N. crassa*, can interact with replication protein A (RPA) in DNA replication (Aalto et al., 2010; Kato et al., 2004). This is the first study to reveal the association between Rdrp protein and DNA replication-related machinery in fungi, suggesting that QDE-1 may participate in nucleic acid synthesis-related reactions at genomic sites related to DNA replication through interaction with RPA, providing support for the regulation of gene expression related to DNA replication (Nolan et al., 2008a). The Rdrp protein in *S. pombe* is involved in DNA replication by participating in heterochromatin formation and maintenance. Rdrp maintains the stability of specific regions of the genome and provides protection for DNA replication (Zofall and Grewal, 2006). Future research should comprehensively investigate Rdrp functions across diverse fungal species to establish a theoretical foundation for understanding its broader roles in both fungi and other eukaryotic organisms.

### 3.4 Other proteins involved in RNAi

Additionally, various other proteins involved in RNA silencing have been identified in eukaryotes. Exportin-5 is responsible for transporting precursor microRNAs (pre-miRNAs) from the nucleus to the cytoplasm (Yi et al., 2003). QIP (QDE-2-interacting protein), an exonuclease discovered in *N. crassa*, specifically cleaves and degrades the passenger strand of siRNAs (Maiti et al., 2007). Similarly, C3PO, an exonuclease found in *Drosophila melanogaster*, performs a comparable function (Liu et al., 2009). HEN1 is involved in the methylation of miRNAs in *A. thaliana* and piRNAs in *D. melanogaster* (Saito et al., 2007; Yu et al., 2005). Moreover, the RITS complex has been found to interact with the RNA-directed RNA polymerase complex (RDRC) and the ARC to mediate heterochromatin silencing (Motamedi et al., 2004; Saito et al., 2007). Whether these components exist in fungi or if analogous factors perform similar functions remains an open question requiring further investigation.

## 4 Research on fungal sRNA biogenesis and biological functions

Significant progress has been made in understanding fungal sRNA biogenesis using *N. crassa* as a model system. During vegetative growth, repetitive transposons and ribosomal DNA loci produce aberrant RNAs (aRNAs) through QDE-1 and QDE-3 (Chang et al., 2012). These single-stranded aRNAs are converted into dsRNA precursors by QDE-1 (Aalto et al., 2010; Kato et al., 2004). In an ATP-dependent process, Dicer unwinds the dsRNA and cleaves it into double-stranded sRNAs with 5′ phosphate, 3′ hydroxyl groups, and 2-nucleotide 3′ overhangs (Pickford et al., 2003).

In the cytoplasm, sRNAs are unwound by an RNA helicase into sense and antisense strands (Cui et al., 2022). The sense strand interacts with mRNA, while the antisense strand binds to and activates the RISC, which contains QDE-2 and QIP (Kato et al., 2004). Once activated, RISC recognizes homologous mRNA transcripts via base pairing and cleaves them 12 nucleotides from the 3′ end of the sRNA, triggering rapid mRNA degradation. This process leads to further degradation of similar mRNA sequences, amplifying the RNAi effect within the fungal cell (Zeng et al., 2024). Additionally, sRNAs not only guide RISC-mediated cleavage but also act as primers for target RNAs. Through an amplification mechanism resembling PCR, Rdrp catalyzes the synthesis of additional dsRNAs, which are subsequently cleaved by Dicer to generate secondary sRNAs, further reinforcing gene silencing (Chang et al., 2012). Nolan et al. (2008a) demonstrated that QDE-1 interacts with RPA, a key component of the DNA replication machinery. Both QDE-1 and RPA are nuclear proteins, and QDE-1 specifically binds to repetitive transgene loci, suggesting that dsRNA synthesis occurs *in situ* using primary transgene transcripts as templates. Similarly, studies in *S. pombe* have shown that the post-transcriptional regulator Mkt1 plays a critical role in post-transcriptional gene silencing and sRNA biogenesis, contributing to the maintenance of heterochromatin (Taglini et al., 2020).

In *N. crassa*, milRNAs are synthesized via four distinct pathways, each requiring different protein components, including Dicers, QDE-2, QIP, RNase III domain-containing proteins, and MRPL3 (Lee et al., 2010). Among these, RNA polymerase III plays a predominant role in milRNA synthesis (Yang et al., 2013). Functionally, fungal milRNAs exhibit similarities to miRNAs in animals and plants, primarily regulating gene expression. Li et al. (2022) performed sRNA sequencing during the infection stage of *F. oxysporum* and identified a QDE-2-dependent milRNA, milR-87, which plays a critical role in fungal pathogenicity. In *N. crassa*, QDE-2 preferentially binds qiRNAs derived from DNA damage sites, while a second Ago protein, Ago2, binds milRNAs. Knockout of QDE-2 does not affect milRNA accumulation, indicating strict functional specialization (Aalto et al., 2010; Pickford et al., 2003). milR-87 regulates virulence by targeting and inhibiting glycoside hydrolase-encoding genes in the pathogen (Li et al., 2022). Similarly, in *Aspergillus flavus*, environmental stress conditions, including varying water activity and temperature, led to differential expression of 135 milRNAs (Bai et al., 2015). These findings suggest that milRNAs may regulate aflatoxin biosynthesis and hyphal growth in response to environmental cues (Bai et al., 2015).

Meiotic silencing by unpaired DNA (MSUD) was first identified in *N. crassa* during the study of the *Ascospore maturation 1* (*asm-1*) gene (Aramayo and Metzenberg, 1996; Shiu et al., 2001). This mechanism occurs during meiotic prophase I when unpaired homologous genes, arising from deletions, duplications, or transpositions, trigger gene silencing. MSUD relies on six core proteins: SAD-1, an RNA-dependent RNA polymerase; DCL-1, a Dicer-like RNase III enzyme; SMS-2, an Argonaute homolog; SAD-2, which localizes SAD-1 to the perinuclear region; QIP, an exonuclease; and SAD-3, a presumed helicase essential for both MSUD and ascospore formation. The *S. pombe* ortholog of SAD-3, Hrr1, is a helicase required for RNAi-induced heterochromatin formation. Both SAD-3 and Hrr1 interact with RNA-dependent RNA polymerase and Argonaute proteins, indicating conserved aspects of silencing complex formation between these fungi (Alexander et al., 2008). During MSUD, unpaired DNA regions generate single-stranded aberrant RNA, which is transported to the perinuclear region and serves as a template for dsRNA synthesis mediated by SAD-1. The dsRNA is subsequently processed by DCL-1 into siRNAs, which guide SMS-2 to recognize and cleave complementary mRNAs. SAD-2 and SAD-3 assist in the localization of SAD-1, ensuring efficient dsRNA production, while QIP removes the passenger strand from the siRNA duplex, facilitating the gene silencing process (Alexander et al., 2008; Shiu et al., 2006; Xiao et al., 2010).

In *M. circinelloides*, RNA silencing mechanisms include both Dicer-dependent and Dicer-independent RNAi pathways (Torres-Martinez and Ruiz-Vazquez, 2016). These pathways not only serve as a defense against foreign nucleic acids but also regulate gene expression through the production of endogenous sRNAs. This regulation enables fungi to modulate specific physiological and developmental processes in response to environmental stimuli. Additionally, *N. crassa*, *C. parasitica*, and *A. nidulans* exhibit antiviral mechanisms akin to those observed in plants, where the presence of exogenous viral RNA triggers RNA silencing-mediated immune responses (Andika et al., 2019). Recent research has revealed that sRNAs play pivotal roles as messengers in plant–pathogenic fungus interactions. On one hand, HIGS influences the infection process of filamentous fungi by targeting fungal virulence genes, thereby reducing pathogenicity. On the other hand, sRNAs secreted by fungi act as effectors that enter plant cells, where they interfere with the expression of host defense-related genes, facilitating fungal infection (Zhao et al., 2021). This cross-kingdom RNA communication underscores the complexity of plant–fungal interactions and highlights the potential for utilizing sRNA-based strategies in disease management.

## 5 Cross-kingdom sRNA trafficking mechanisms between fungi and plants

**Figure 3:**
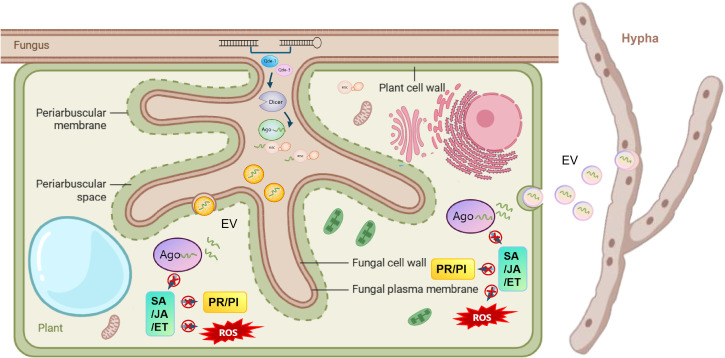
Fungal small RNA cross-kingdom trafficking routes. This schematic depicts the bidirectional sRNA-mediated communication between a fungus and a plant cell. Fungal sRNA delivery: Fungal hyphae secrete extracellular vesicles (EVs) carrying sRNAs (bound to AGO proteins) into the plant apoplast. These EVs are internalized into the plant cell; fungal sRNA-mediated plant immune suppression: fungal sRNAs hijack the plant’s RNAi machinery (Dicer/AGO) to silence plant defense-related molecules, including the following: PR/PI: pathogenesis-related proteins/protease inhibitors; SA/JA/ET: key defense hormone signaling pathways; ROS: reactive oxygen species (oxidative burst). Plant sRNA counterdefense: Plant-derived sRNAs (also via EVs) can be taken up by fungal hyphae to target fungal virulence genes, though this pathway is less prominent in this diagram. The interface (periarbuscular membrane/space) between fungal and plant cells facilitates sRNA exchange during the interaction.

Cross-kingdom RNA interference has emerged as a key regulatory axis in plant–fungal interactions, where sRNAs act as mobile signaling molecules to mediate bidirectional communication (**Table 3**). Fungal sRNAs translocate into plant cells to suppress host immunity, while plant-derived sRNAs target fungal virulence genes to restrict infection. The trafficking of these sRNAs across kingdom boundaries involves conserved molecular mechanisms, species-specific adaptations, and complex regulatory networks, which are critical for understanding plant–fungal coevolution and developing RNAi-based biocontrol strategies (**Figure 3**).

**Table 3:** Functions of fungal small RNAs in biocontrol interactions.

Fungal biocontrol agent (BCA)	sRNA type	sRNA target motif (5′/3′)	Function	Target organism/Gene	Delivery mechanism	Reference
*Clonostachys rosea*	cro-mir-1, cro-mir-2, cro-mir-3, cro-mir-4, cro-mir-5, cro-mir-6, cro-mir-8, cro-mir-9, cro-mir-11, cro-mir-13, cro-mir-34, cro-mir-36, cro-mir-72	5′-UUG (core motif)	Suppress pathogen virulence by cleaving mRNA of pathogenicity genes; inhibit hyphal elongation and spore germination	*F. graminearum*/FGSG_9686, FGSG_00376, FGSG_02083, FGSG_04181, FGSG_06384, FGSG_07067, FGSG_07665, FGSG_08359, FGSG_08915, FGSG_11033, FGSG_11973, FGSG_13747	Natural secretion (extracellular vesicles/RBPs)	Piombo et al. (2021)
*Beauveria bassiana*	bba-milR1	3′-AAG (seed region)	Suppress insect innate immunity by silencing the Toll pathway; reduce melanization and antimicrobial peptide production	*Anopheles stephensi* (mosquito)/*spz4*, CLIPB9	Natural secretion (hyphal exudates)	Cui et al. (2019)
*Pythium periplocum*	ppe_mir_1, ppe_mir_2	5′-UAU (conserved motif)	Predicted to target virulence genes via translational repression; inhibit appressorium formation and host penetration	*B. cinerea*/BCIN_07g03380, BCIN_14g01020, Bccch1, BCIN_08g04100, BCIN_04g03280 *P. infestans*/PITG_00939, PITG_03209, PITG_13437	Natural secretion (apoplastic diffusion)	Piombo et al. (2023), Piombo et al, 2021), Taglini et al. (2020)
*Trichoderma harzianum*	tha-milR10/tha-siRNA23	3′-Hydroxyl group (unmodified)	Inhibit mycorrhizal colonization by silencing G-protein signaling; reduce hyphopodium formation and arbuscule development	*Fusarium oxysporum* f. sp. *lycopersici*: FoSIX6 (effector) FoCWDE2 (cellulase) FoMAPK2 (signaling); *Verticillium dahliae*: VdSCP1 (effector) VdPR1 (protease)	Spray-induced (root drenching)	Carreras-Villasenor et al. (2013); Nolan et al. (2008a)
*Metarhizium anisopliae*	mae-siRNA45/mae-milR7	5′-Uracil (fungal sRNA signature)	Dual mechanism: silence fungal pathogen virulence genes; upregulate plant JA/SA signaling	*Spodoptera frugiperda*: SfCYP6B46 (detoxification enzyme) SfGST1 (glutathione S-transferase); *Metarhizium anisopliae*: MaMAD1 (adhesin) MaPTH1 (protease)	Natural secretion (hyphal exudates + EVs); root colonization	Chen and Rechavi (2022); Li et al. (2022)

### 5.1 Core trafficking routes of fungal sRNAs into plant cells

Fungal pathogens have evolved multiple specialized pathways to deliver sRNAs into host plant cells, ensuring efficient hijacking of the plant RNAi machinery. These routes are shaped by the pathogen’s lifestyle and the specific plant–fungal interaction context (Zhao and Guo, 2022).

#### 5.1.1 Extracellular vesicle-mediated trafficking

Extracellular vesicles are membrane-bound nanoparticles that serve as universal carriers for sRNAs, proteins, and lipids in cross-kingdom communication. This pathway is predominantly utilized by necrotrophic and hemibiotrophic fungi during active infection. 1) EV biogenesis and loading: Fungi synthesize EVs in the Golgi apparatus or via plasma membrane budding, with tetraspanin proteins acting as key biomarkers and sorting factors (Cai et al., 2019; He et al., 2023). Fungal sRNAs are selectively loaded into EVs through RNA-binding proteins (RBPs) or direct interaction with membrane lipids, ensuring protection from extracellular RNases in the plant apoplast (Weiberg et al., 2013). 2) Plant cell uptake: Fungal EVs are internalized by plant cells via clathrin-mediated endocytosis (CME) or lipid raft-dependent pathways. In *B. cinerea*–*Arabidopsis* interactions, EVs carrying Bc-sRNA3.1 and Bc-sRNA4.2 bind to plant clathrin light chain 1 at the infection site, facilitating vesicle fusion with the plant plasma membrane. Mutations in plant CME genes reduce EV uptake by 60% and significantly enhance plant resistance to *B. cinerea*, confirming the functional importance of this route (Weiberg et al., 2013). 3) Species-specific adaptations: EV-mediated trafficking is particularly critical for necrotrophs like *B. cinerea*, which encounter harsh apoplastic environments rich in RNases during host tissue necrosis (He et al., 2023). EV membranes shield sRNAs from degradation, ensuring their stable delivery to plant cells. Comparative studies show that EVs from *M. oryzae* are enriched in sRNAs targeting plant pattern recognition receptor (PRR) genes (Raman et al., 2017), while EVs from *Verticillium dahliae* carry sRNAs that suppress plant jasmonic acid (JA) signaling (Zhao et al., 2021).

#### 5.1.2 RNA-binding protein-mediated trafficking

For hemibiotrophic fungi that establish long-term biotrophic interactions with plants, RBPs serve as sRNA carriers to mediate EV-independent trafficking. 1) RBP–sRNA complex formation: Fungal RBPs contain conserved RNA-recognition motifs (RRMs) that bind specifically to sRNAs with 5′-uracil (U) residues, a common feature of fungal sRNAs involved in cross-kingdom silencing. These complexes form in the fungal cytoplasm and are secreted into the apoplast via unknown transporters, possibly involving ABC transporters or plasma membrane channels (Piombo et al., 2023; Wang et al., 2022). 2) Stabilization and delivery: RBPs protect sRNAs from apoplastic RNases through direct binding, maintaining sRNA integrity during translocation. In *F. graminearum*–wheat interactions, FgRbp1–sRNA complexes bind to wheat plasma membrane-localized receptor-like kinases (RLKs), triggering endocytosis and release of sRNAs into the plant cytoplasm. Unlike EVs, RBP–sRNA complexes are smaller and can penetrate plant cell walls via plasmodesmata, enabling sRNA delivery to adjacent uninfected cells (Wang et al., 2022). 3) Functional validation: Knockout of FgRbp1 in *F. graminearum* reduces fungal sRNA accumulation in wheat cells by 75% and attenuates pathogen virulence, while complementation with wild-type FgRbp1 restores sRNA trafficking and pathogenicity. Similar RBPs have been identified in *F. oxysporum* and *Colletotrichum higginsianum*, indicating conservation of this pathway among hemibiotrophic fungi (Song et al., 2018).

#### 5.1.3 Direct diffusion via plasmodesmata

In biotrophic interactions where fungi form intimate hyphal structures, sRNAs can diffuse directly from fungal hyphae into plant cells via plasmodesmata. 1) Physical connection facilitation: Biotrophic fungi establish specialized interfaces that connect fungal hyphae to plant cells, reducing the physical barrier for sRNA translocation. Plasmodesmata at these interfaces are modified to allow passage of small molecules (≤10 kDa), including sRNAs and RBPs (Zhao et al., 2021). 2) sRNA selectivity: Direct diffusion is not random; fungal sRNAs are enriched in plasmodesmata through interaction with plant chaperone proteins that facilitate their transport. In *Blumeria graminis*–barley interactions, fungal milRNAs are detected in plant cells adjacent to haustoria, and inhibition of plasmodesmata function reduces sRNA accumulation by 80% (Chand et al., 2017).

### 5.2 Mechanisms of plant sRNA trafficking to fungal cells

Plants have evolved counterdefense mechanisms to deliver sRNAs into fungal pathogens, targeting key virulence factors to inhibit infection. This reciprocal trafficking pathway is less well-characterized but involves conserved secretion and uptake mechanisms.

#### 5.2.1 Plant EV-mediated secretion

Plants secrete EVs from root and leaf cells that carry sRNAs targeting fungal genes. These plant EVs are internalized by fungal hyphae via endocytosis. 1) Plant EV biogenesis: Plant EVs are derived from the trans-Golgi network or plasma membrane, with *Arabidopsis* AtVAMP727 (a vesicle-associated membrane protein) playing a key role in sRNA loading. Plant sRNAs are selected for EV packaging based on their 3′-methylation status (mediated by HEN1) and interaction with plant RBPs (Yu et al., 2005). 2) Fungal uptake and silencing: In *Arabidopsis*–*Fusarium oxysporum* interactions, plant EVs carrying sRNAs targeting fungal cell wall-degrading enzymes (CWDEs) are internalized by fungal hyphae via clathrin-dependent endocytosis. These sRNAs are loaded into fungal Argonaute proteins, leading to silencing of FgCWDE1 and FgCWDE2 and reduced fungal pathogenicity (Song et al., 2018).

#### 5.2.2 Apoplastic diffusion and fungal internalization

Plant sRNAs can also be secreted directly into the apoplast via ABC transporters, followed by active uptake by fungal cells. 1) Apoplastic secretion: Plant sRNAs are transported across the plasma membrane by ABC transporters, which have broad substrate specificity for small nucleic acids. This secretion is induced upon fungal infection, with sRNA levels in the apoplast increasing 5–10-fold within 24 h of inoculation (Zhao et al., 2021). 2) Fungal uptake mechanisms: Fungi internalize apoplastic sRNAs via plasma membrane-localized RBPs that bind to plant sRNAs and mediate their transport into the cytoplasm. In rice–*M. oryzae* interactions, rice sRNAs targeting fungal effector genes are taken up by fungal hyphae, leading to reduced effector expression and enhanced rice resistance (Raman et al., 2017).

### 5.3 Key molecular determinants of cross-kingdom sRNA trafficking

The specificity and efficiency of sRNA trafficking are governed by conserved molecular determinants, including sRNA structural features, chaperone proteins, and host–pathogen interface components.

#### 5.3.1 Chaperone proteins and RNA-binding factors

RBPs and chaperone proteins play dual roles in sRNA stabilization and trafficking, acting as escort molecules across kingdom boundaries. 1) Fungal RBPs: As discussed earlier, fungal RBPs bind sRNAs to protect them from RNases and mediate interactions with plant cell surface receptors. These RBPs often contain nuclear localization signals (NLSs) that facilitate sRNA delivery to the plant nucleus, where they silence transcription of immune genes (Wang et al., 2022; Weiberg et al., 2013). 2) Plant chaperones: Plant HSP70 and HSP90 proteins interact with fungal sRNAs in the apoplast, enhancing their solubility and facilitating EV uptake. In *Arabidopsis*, AtHSP70 binds to *B. cinerea* sRNAs, increasing their affinity for clathrin and promoting endocytosis (Chand et al., 2017).

#### 5.3.2 Host–pathogen interface components

The physical interface between fungi and plants provides critical cues for sRNA trafficking, with cell wall components and membrane receptors acting as key regulators. 1) Cell wall modifications: Fungal pathogens secrete cell wall-degrading enzymes to loosen plant cell walls, creating channels for sRNA diffusion. Plant cells respond by depositing callose at the infection site, which can block sRNA trafficking—highlighting a dynamic evolutionary arms race (Raman et al., 2017). 2) Membrane receptors: Plant plasma membrane receptors and fungal membrane proteins mediate specific recognition of EVs or RBP–sRNA complexes, ensuring targeted sRNA delivery. In tomato–*B. cinerea* interactions, tomato RLK3 binds to BcPls1 on fungal EVs, triggering CME and EV internalization (Weiberg et al., 2013).

## 6 Applications of sRNA in fungi

The current applications of fungal sRNAs are primarily focused on fungal and plant interactions. Qiao et al. (2021) developed dsRNAs targeting key pathogenic genes of seven plant–pathogenic fungi and oomycetes and evaluated their effectiveness using SIGS. The results demonstrated significant inhibition of infections caused by *B. cinerea*, *S. sclerotiorum*, *Rhizoctonia solani*, *A. niger*, *V. dahliae*, and *Phakopsora pachyrhizi* (Ouyang et al., 2024; Piombo et al., 2024) Similarly, HIGS has been successfully employed to express sRNAs targeting six pathogenicity-related genes in *M. oryzae*, conferring resistance to rice blast disease in rice plants (Wang and Dean, 2022). These findings highlight the potential of HIGS and exogenous sRNA-based inhibitors as promising strategies for managing fungal diseases. By regulating the expression of sRNAs or their target genes, these approaches may revolutionize plant protection and open new avenues for agricultural biotechnology.

A core mechanism underlying these sRNA-mediated plant–fungal interaction regulations lies in the modulation of plant-specific immune signaling pathways (salicylic acid, JA, and ethylene pathways) by fungal sRNAs. Fungal pathogens can secrete sRNAs into plant cells to hijack host immune signaling, while beneficial fungi or engineered sRNAs can also be used to rewire these pathways for enhanced plant defense.

Salicylic acid (SA) signaling is central to plant defense against biotrophic pathogens, and fungal sRNAs often target key components of this pathway to suppress immunity. *Botrytis cinerea* secretes Bc-sRNA3.1, which directly targets the plant gene AtNPR1 in *Arabidopsis*, reducing SA accumulation and inhibiting the expression of downstream pathogenesis-related (PR) genes (Weiberg et al., 2013). In contrast, HIGS-expressed sRNAs targeting fungal sRNA biogenesis genes can restore plant SA pathway activity, enhancing resistance to necrotrophic pathogens (Qiao et al., 2021). Additionally, the ectomycorrhizal fungus *Pisolithus microcarpus* secretes Pmic_miR-8, which modulates host SA signaling in *Eucalyptus grandis* roots by silencing SA-responsive transcription factors, thereby stabilizing the symbiotic interaction (Piombo et al., 2023).

The JA pathway mediates defense against necrotrophic fungi and herbivores, and fungal sRNAs have evolved to disrupt this cascade. *Verticillium dahliae* sRNAs target tomato *SlJAZ2* and *SlMYC2* genes, which encode core JA signaling repressors and activators, respectively, altering JA signal transduction to promote fungal colonization (Zhao et al., 2021). Field trials of SIGS using dsRNAs targeting Vd-sRNA136 precursor genes showed restored JA pathway activation in cotton, reducing *Verticillium* wilt incidence by 45% (Ouyang et al., 2024). For beneficial interactions, arbuscular mycorrhizal (AM) fungi secrete sRNAs that upregulate JA-responsive defense genes in host roots, priming systemic resistance against subsequent pathogen attack (Fan et al., 2025).

Ethylene (ET) synergizes with JA to regulate plant defense and development, and fungal sRNAs interfere with this pathway by targeting ET biosynthesis or signaling genes. *Fusarium graminearum* secretes Fg-sRNA16, which silences wheat *ACS2* (a key gene for ET biosynthesis), reducing ET production and weakening host cell wall reinforcement (Wang et al., 2022). HIGS constructs expressing sRNAs complementary to Fg-sRNA16 have been shown to recover wheat ET levels and enhance resistance to *Fusarium* head blight (Fan et al., 2025). Meanwhile, *M. oryzae* sRNAs target rice EIN2 (an ET signaling transducer), suppressing ET-mediated hypersensitive response and facilitating blast fungus invasion (Raman et al., 2017).

Fungal cross-kingdom RNA plays important roles in the interactions between fungi and their hosts. These research findings demonstrate that fungal cross-kingdom RNA plays a crucial role in the interactions between fungi and their hosts, providing new insights into the molecular mechanisms of host–pathogen interactions. Moreover, they offer potential targets and strategies for the development of novel pest control methods and crop protection technologies. Based on the results of co-expression analysis, some virulence effector genes were determined as potential RNAi targets. Interfering with the expression of these genes may disrupt the pathogenic mechanism of the pathogen, thus achieving the purpose of controlling soybean rust.

## 7 Prospects

sRNAs exert pivotal regulatory functions across all eukaryotic organisms, yet critical knowledge gaps persist in fungal sRNA research. First, it remains to be clarified whether novel classes of fungal sRNAs exist beyond the currently identified subtypes. While preliminary evidence has confirmed that sRNAs modulate core fungal biological processes—including growth, development, and stress adaptation—the precise molecular mechanisms underlying their activity are far from fully elucidated. Key unresolved questions include defining the context-specific functions of individual sRNA molecules, characterizing their spatiotemporal expression patterns, dissecting the structural dynamics and functional specificities of sRNA-associated protein complexes, and unraveling the evolutionary drivers that have shaped the diversity of fungal sRNA pathways (Chand et al., 2017).

Technical bottlenecks continue to hinder the comprehensive study of fungal sRNAs. Although advancements have been made in detecting, isolating, and characterizing fungal sRNAs, limitations such as low sensitivity for detecting low-abundance sRNA species and the technical complexity of purifying structurally modified sRNAs restrict the scope of exploratory studies. Moreover, functional validation of fungal sRNAs is hindered by the recalcitrance of many fungal species to efficient genetic transformation. The lack of robust protocols for targeted gene knockout or *in vivo* overexpression in non-model fungi compromises the accuracy and reproducibility of sRNA functional assays (Hamby et al., 2020). Compounding these challenges is the extreme intricacy of fungal sRNA regulatory networks, involving numerous interactions between sRNAs, their target mRNAs, and downstream signaling cascades. Unraveling these interactions presents a major challenge for researchers aiming to elucidate the full scope of sRNA-mediated gene regulation.

Nevertheless, with the rapid advancement of computational science, bioinformatics, and biotechnology, biological research has entered the era of whole-genome studies. It is anticipated that the regulatory mechanisms governing fungal sRNA biogenesis and function will be progressively elucidated in the near future. Additionally, understanding fungal sRNA silencing mechanisms may facilitate the development of a novel classification system based on RNA silencing modalities, offering new insights into the evolutionary relationships among fungi and between fungi, animals, and plants. Given their intimate link with fungal growth, development, pathogenicity, and metabolism, sRNAs are expected to play increasingly significant roles in pathogen control and genetic engineering. Future research may leverage sRNA-based technologies to enhance biocontrol strategies and improve the efficiency of fungal strains used in biotechnology and industrial applications.
